# The Association between Kidney Function Biomarkers and Delayed Memory Impairments among Older Adults in the European North of Russia

**DOI:** 10.3390/brainsci13121664

**Published:** 2023-11-30

**Authors:** Liliya Poskotinova, Anna Kontsevaya, Alexander V. Kudryavtsev

**Affiliations:** 1 Biorhythmology Laboratory of the Institute of Environmental Physiology, N. Laverov Federal Center for Integrated Arctic Research of the Ural Branch of the Russian Academy of Sciences, 163001 Arkhangelsk, Russia; liliya200572@mail.ru; 2 Central Scientific Research Laboratory, Northern State Medical University, 163069 Arkhangelsk, Russia; 3 Department of Public Health, National Medical Research Centre for Therapy and Preventive Medicine, 101000 Moscow, Russia; koncanna@yandex.ru; 4 Department of Community Medicine, UiT The Arctic University of Norway, N-9037 Tromsø, Norway

**Keywords:** aging, delayed memory, kidney function, intrinsic capacity

## Abstract

The prevention of memory decline requires better knowledge of biological markers. We studied the associations between kidney function biomarkers and memory decline (assessed with the Mini-Mental State Examination—MMSE) in elderly individuals without dementia (MMSE 24–30, age 60–74 years, *n* = 643, Arkhangelsk, Russia). Participants were divided by sex and into three groups according to the delayed memory performance: recall of 0–1, 2, and 3 out of 3 words. The median of serum creatinine was 82 μmol/L in men who recalled 2 words and both medians in those recalling 3 and 0–1 words were 87 μmol/L. The 90th percentile for creatinine in men recalling 0–1 words (115.0 μmol/L) exceeded the upper limit of the normal range (110.5 μmol/L), while those who recalled 3 and 2 words had 90th percentiles within the normal range (109 and 101 μmol/L, respectively). Glomerular filtration rates were normal (≥60 mL/min/1.73 m^2^) with a median of 92.0 mL/min/1.73 m^2^ in men who recalled 2 words, 84.4 and 84.9 mL/min/1.73 m^2^ in men who recalled 3 and 0–1 words, respectively. None of these associations were observed in women. A reduced serum creatinine in older non-demented men may indicate the initial stages of memory decline, while the increased creatinine may reflect further stages of memory impairment.

## 1. Introduction

Aging leads to functional decreases in all systems of the human body, including cognitive functions such as memory, speech, gnosis, praxis, visual-constructive skills, et cetera. Memory decline is the most frequent complaint in older adults at the predementia stage, which, until recently, has been referred to as “age-related memory impairment”, but only in half of patients with minor memory impairments are these cognitive deficits detected via neuropsychological methods [1]. Cognitive decline at the predementia level (minor cognitive impairment) in the elderly can last for a long time [2], and there is a need for biomarkers allowing the differentiation between a minor age-related cognitive decline and the first stages of dementia, which significantly affects the quality of life in subsequent years. Various neuropsychological tests assess delayed memory function by measuring a patient’s ability to recall a certain numbers of words after a given time, or after having another cognitive task to complete between the listing of the words and their recall. For example, the Montreal Cognitive Assessment, which is used for detecting both predementia and severe cognitive impairment, contains a subtest for the delayed recall of five words [3]. It has been shown that poor performance in the delayed memory test is associated with a decrease in the neural activity of the hippocampus and frontal cortex [4]. The Mini-Cog test contains a subtest to assess delayed memory by asking participants to recall three words, but this test is only used to screen for dementia [5]. The Mini-Mental State Examination (MMSE) also contains a subtest for delayed memory assessment, which is based on the recall of three words after their prior repetition and another cognitive task in between [6,7]. This MMSE subtest can be used as an independent diagnostic tool. For example, it has been described that the prognosis of delirium after surgery was dependent on the preoperative delayed memory performance according to the MMSE: for each of the three words not recalled, the probability of delirium increased by 50% [8].

Research into biochemical and cytogenetic biomarkers of age-related memory decline is currently ongoing in animal species. For example, a recent study on mice by Orock et al. (2020) described the relationship between age and the decreased release of the vesicular receptor protein synaptobrevin-2, which is expressed in cortical and hippocampal neurons and is necessary for synaptic transmission. The authors suggested that age-related decrease in the vesicular release of this protein in neurons causes the impairment of synaptic plasticity, affecting memory and learning processes [9]. It was shown that the enzyme mitochondrial aconitase-1 regulates age-related memory deterioration in Drosophila through autophagy/mitophagy-mediated neuronal plasticity [10]. In the absence of similar studies on humans, peripheral blood parameters associated with both somatic function and cognitive impairment can potentially serve as early biomarkers of memory impairment in elderly people. Serum-based kidney function indicators may be such biomarkers. Serum levels of creatinine and uric acid and glomerular filtration rate (GFR) reflect kidney function. A significant excess of normal creatinine values and a reduction in GFR indicate renal insufficiency [11]. Low creatinine levels may indicate decreased efficiency of protein metabolism and the risk of sarcopenia, especially in elderly people [12,13]. There are data supporting the relationship between serum creatinine level and cognitive function, but this relationship was described as nonlinear, i.e., both low and high creatinine levels can be considered as the metabolic basis for cognitive disorders [14,15]. Residents of Russia’s Arctic territories tend to have a traditional diet that is rich in protein-lipid components, which continues to prevail regardless of the recent shifts towards increased carbohydrates consumption [16]. For this reason, the activity of the enzyme systems that are responsible for protein synthesis and the excretion of its metabolites remain high. This is reflected in the significantly higher levels of creatinine in inhabitants of the European North of Russia, compared to residents of the southern regions of Russia and neighboring countries, with relatively high serum creatinine concentrations commonly revealed in combination with increased adrenaline and histamine levels [17].

Due to the growing life expectancy, the concept of healthy aging is increasingly used in relation to the concept of the intrinsic capacity as a set of locomotor, sensory, cognitive, and psychological capabilities, which are the key components of quality of life in older ages [18]. Respectively, conventional disease biomarkers are being reconsidered in relation to preserving health and functional abilities [19]. The tools used for measuring functional abilities and calculating the composite indices of the intrinsic capacity are not yet standardized and are being replenished [20]. One of the challenges is to define the optimal levels of various biomarkers for maintaining intrinsic capacity in elderly people who live in different climatic, geographical, and socio-economic conditions. In the European North of Russia, the approach of maintaining the intrinsic capacity as the basis for healthy aging has been scarcely used. This may partly be because of the limited knowledge of the biomarkers of preserved functional abilities in the elderly in this region.

The leading hypothesis in this study was that the serum concentrations of kidney function biomarkers might serve as indicators of delayed memory function in the elderly people with intact cognitive function or predementia who reside in northern areas. Accordingly, the aim of this study was to assess the association between serum levels of creatinine, uric acid, and GFR and delayed memory function in a population sample of elderly people without dementia living in the European North of Russia.

## 2. Materials and Methods

In 2021, 1348 men and women aged 40 to 74 years were examined in Arkhangelsk as a part of the Russian multi-center cross-sectional study “Epidemiology of cardiovascular diseases and their risk factors in the regions of the Russian Federation. The Third Study (ESSE-RF3)”. These participants were Arkhangelsk residents who had previously been included in the random population sample of the “Know Your Heart” study (2015–2017, *n* = 2380), recruited using a depersonalized database of the territorial mandatory health insurance fund, which contained the addresses of all the insured residents of Arkhangelsk aged 35–69 years, which was supplemented with sex and age information. Random addresses were selected for visits, and men and women of appropriate sex and age (±2 years) living at those addresses were invited to participate in the study. The response proportion in the “Know Your Heart” study was 68%. In 2021, to recruit ESSE-RF3 participants, receptionists at the Consultative and Diagnostic Polyclinic of the Northern State Medical University (CDP NSMU) made telephone calls to 2258 “Know Your Heart” participants who had previously provided their contact information, consented to be invited to participate in new studies, and were 40–74 years old at the time of recruitment. The response proportion was 60%. The sampling methods used for the ESSE-RF3 in the Arkhangelsk region has been described in more detail in earlier publications based on the study [21,22].

An examination of ESSE-RF3 participants was conducted at CDP NSMU and included the Mini-Mental State Examination (MMSE), blood sampling, blood pressure measurement, anthropometry, and a standardized questionnaire collecting data on socio-demographic characteristics, lifestyle, and doctor-diagnosed diseases. MMSE scores <24 indicate dementia, scores of 24–27 indicate predementia, and scores of 28–30 indicate intact cognitive function [23]. For this study, we selected all 676 ESSE-RF3 participants in the age range of 60 to 74 years. Three participants with incomplete MMSE data and 30 participants with significant cognitive impairments (MMSE score < 24) were excluded. The analyzed sample comprised 643 participants from the ESSE-RF3 study. The selected participants were divided by sex and by the results of the MMSE assessment of the delayed recall of 3 words. The recall test was performed as follows: first, the participants were asked to repeat three words after the tester without being told they had to memorize the words, then the attention–calculation test was carried, and after that, the participants were asked to recall the three words. For each sex, subgroup 1 comprised participants with no evidence of delayed memory impairments (recall of 3 words), subgroup 2 included participants with reduced delayed memory (recall of 2 words), and subgroup 3 consisted of those with further impaired delayed memory (recall of 0–1 words).

Blood samples were collected via venipuncture after overnight fasting and were stored at room temperature for 30 min for clot formation. Within 2 h after venipuncture, the samples were centrifuged for 15 min at 3500 rpm and at 4 °C for serum separation. Serum samples were aliquoted into cryovials and frozen at −25 °C until assayed. The studied kidney function parameters were serum creatinine concentration (μmol/L), serum uric acid level (mg/dL), and GFR (ml/min/1.73 m^2^). Creatinine and uric acid levels were measured at the ESSE-RF3 laboratory located at the National Medical Research Center for Therapy and Preventive Medicine (Moscow, Russia), where frozen serum samples had been shipped from Arkhangelsk on dry ice. The colorimetric method was applied. Creatinine and uric acid levels were measured in blood serum on the Architect 8000 biochemical analyzer (USA), using Abbott reagents. Normative creatinine values were set in the range of 50.4–110.5 μmol/L. Normative uric acid values ranged from 3.5 to 7.2 mg/dL (in men) and 2.6–6.0 mg/dL (in women).

The estimated GFR based on the serum creatinine concentration (eGFRCr) was calculated using the 2021 CKD-EPI Creatinine Equation (2021), as recommended for adults by the National Kidney Foundation [24]. The formula used was: eGFR_cr_ = 142 × min(S_cr_/κ, 1)^α^ × max(S_cr_/κ, 1)^−1.200^ × 0.9938^Age^ × 1.012 [if female], where S_cr_ = standardised serum creatinine in mg/dL, κ = 0.7 (female) or 0.9 (male), α = −0.241 (female) or −0.302 (male), min(S_cr_/κ, 1) is the minimum of S_cr_/κ or 1.0, max(S_cr_/κ, 1) is the maximum of S_cr_/κ or 1.0, and Age = age in years. A reduction in eGFRcr was considered clinically significant at <60 mL/min/1.73 m^2^, as recommended for adults over 18 years [25].

As we studied a random population-based sample, there were no indications that creatinine, eGFRCr, and serum uric acid levels were biased markers of kidney function because of specific hydration features and special diets of the study participants.

Socio-demographic (age, education), lifestyle (smoking, alcohol consumption), anamnestic (self-reports of doctor-diagnosed arterial hypertension, diabetes mellitus, chronic liver and kidney diseases), and anthropometric (body mass index) characteristics, which could be associated with both renal and cognitive functions, were considered as potential confounders.

Absolute values (Abs) and percentages (%) were used to present categorical variables. Means (M) with standard deviations (±SD) and/or medians (Me) with 10th and 90th percentiles (10p–90p) were used to present continuous variables. Normality of the data was checked by visual assessment and using Shapiro–Wilk test. Continuous variables with skewed distributions were analyzed in ln-transformed form. We compared men and women based on socio-demographic, lifestyle, health, and cognitive characteristics using Pearson’s χ^2^ test for categorical variables and *t*-test for independent samples for continuous variables. The identified differences guided further sex-stratified analyses. The subgroups of men and women, defined by the number of words recalled at the delayed memory test, were compared based on socio-demographic, lifestyle, and health characteristics using Pearson’s χ^2^ test for categorical variables and analysis of variance (ANOVA) for continuous variables. The subgroups were also compared based on kidney function biomarkers with adjustments for age in women and for age and hypertension in men. The adjustment for age in both sexes was a priori, while the decision to adjust for hypertension in men was based on findings in the preceding analyses. The adjusted comparisons were made using analysis of covariance (ANCOVA). Linear and quadratic trends in kidney function biomarkers across the three subgroups of men and women (subgroup number entered as a continuous independent variable with corresponding values 1–3) were estimated with the same adjustments and using multivariable linear regressions without and with quadratic terms. Statistical analyses were performed using STATA 17.0 software (StataCorp, College Station, TX, USA).

## 3. Results

The studied sample comprised 381 (59.3%) women and 262 (40.7%) men. The mean age of women (66.9 years) was slightly higher than that of men (66.2 years) (Table 1). Compared to men, women tended to include a larger percentage of participants with a secondary professional education, but differences in the distribution of educational attainments between men and women were insignificant. The percentage of smokers and the frequency of alcohol drinking were higher in men, and the body mass index was, on average, higher in women. Compared with men, women more commonly self-reported arterial hypertension (85.3% and 77.9%, respectively), liver disease (13.2% and 3.1%), and kidney disease (16.0% and 9.5%).

According to the results of the MMSE test, the proportion of participants with predementia was higher in men (40.1%) than in women (32.6%). Of all the MMSE subtests, mistakes were most common in the delayed memory (word recall) subtest assignments, and were more often seen in men (67.2%) compared with women (54.3%). Men also included a higher percentage of participants who made mistakes in the reading and writing subtest (19.1% in men vs. 12.6%, in women).

Among women, 174 (45.7%) had no evidence of delayed memory impairments (recalled three words), 158 (41.5%) had reduced delayed memory (recalled two words), and 49 (12.9%) had further impaired delayed memory (recalled 0–1 words) (Table 2). Socio-demographic, lifestyle, and health characteristics of women did not differ significantly between the subgroups with varying numbers of words recalled.

Men had significantly worse performances in the delayed memory test, compared with women (*p* < 0.001). Among them, 86 (32.8%) recalled three words, 114 (43.5%) recalled two words, and 62 (23.7%) recalled one or none of the words (Table 3). The presence of arterial hypertension was more frequently reported by men who recalled 0–1 words (90.3%) compared with men who recalled 2 and 3 words (71.9% and 76.7%, respectively).

In women, the total MMSE score decreased from subgroup 1 to subgroup 3 (Table 4) along with the increasing proportion of participants with predementia (19.5%, 37.3%, and 63.3%, respectively; *p* < 0.001). However, creatinine, uric acid, and eGFRcr levels did not differ significantly between the three subgroups. The 90th percentile for uric acid values in all three subgroups (7.3–7.4 mg/dL) slightly exceeded the normative range (up to 6.0 mg/dL). The 10th percentile for eGFRcr was below the normative level (<60 mL/min/1.73 m^2^) in women who recalled two words (58.1 mL/min/1.73 m^2^).

In men, similarly to women, the total MMSE score decreased from subgroup 1 to subgroup 3 (Table 5) along with the increasing proportion of participants with predementia (19.7%, 39.5%, and 69.4%, respectively; *p* < 0.001).

The distribution of median creatinine values across the three subgroups of men was U-shaped: the lowest median (82 μmol/L) was in those who recalled 2 words, and the medians were equal (87 μmol/L) in those who recalled 3 and 0–1 words. This was confirmed by the presence of a quadratic relationship (*p*_quadratic trend_ = 0.002) between the number of recalled words and the creatinine level. The 90th percentile value for the creatinine level in men who recalled 0–1 words (115.0 μmol/L) exceeded the upper limit of the normal range (110.5 μmol/L), while those who recalled 3 and 2 words had their 90th percentile values (109 and 101 μmol/L, respectively) within the normal range.

Similarly to women, the 90th percentile for uric acid values in all three subgroups of men was above the upper limit of the normal range (up to 7.2 mg/dL). The eGFRcr had an inverse U-shaped distribution across the three subgroups of men (*p*_quadratic trend_ = 0.005). The highest median (92 mL/min/1.73 m^2^) was in those who recalled 2 words, and the medians were almost equal in those who recalled 3 and 0–1 words (84.4 and 84.0 mL/min/1.73 m^2^, respectively). The observed eGFRcr levels reflected preserved renal filtration function in all three subgroups.

## 4. Discussion

The study demonstrated non-linear associations of the levels of creatinine and eGFRcr with delayed memory function in elderly men without significant cognitive decline who reside in the European North of Russia. Reduced serum creatinine levels and increased GFR in older men were found to be associated with the initial but not with the more advanced stages of delayed memory impairments.

Delayed word recall was the most frequent cognitive impairment in the Russian sample of elderly people without dementia, indicating the predominance of the amnestic monofunctional type (with selective memory impairment) of mild cognitive impairment [2,26]. The prevalence of delayed memory impairments was higher in elderly men than in elderly women with no dementia, even with a slightly higher mean age of women, and this was consistent with the higher proportion of men at the predementia stage (MMSE score of 24–27). Earlier research findings on the effects of sex differences in the manifestation of mild cognitive impairment in an older age are controversial. For example, analysis of sex differences in mild cognitive impairment among elderly inhabitants of the United Kingdom (mean age 63 years) has demonstrated a stronger association between mild cognitive impairments and subsequent cognitive decline in men than in women [27]. In a Swedish population sample aged 60 years or older, the manifestation of mild cognitive impairment was not found to be associated with sex [28]. No sex differences in the severity of cognitive impairment were also found among Chinese residents aged 65–79 years, but these differences were observed in the age group above 80 years (more pronounced impairment was observed in women) [29]. Therefore, the effects sex differences in the manifestation of age-related cognitive impairments may vary across settings.

In our study, the subgroups of men and women with varying delayed memory performance did not differ in age or education. Smoking and frequency of alcohol consumption were not associated with delayed memory in both women and men. This might have indicated little age dependence of the initial memory impairments if taken in the age span of 60–74 years. With respect to education, smoking, and alcohol, the absence of the associations could be a reflection of other factors being the drivers of the initial stages of memory decline in the studied population.

The most common comorbidity for the impaired delayed memory in men was arterial hypertension. Although being reported by a smaller percentage of men, compared with women, only in men was hypertension associated with poor delayed memory, being most prevalent (90.3%) in those with 0–1 word recall. The significance of hypertension as a comorbid condition aggravating memory decline has been shown in young and middle-aged patients with kidney pathology [30]. Alternative explanations for this finding might be the differential reporting of hypertension by men and women and a lower compliance with treatment in men. As a consequence, cerebral hemodynamics and neurotransmitter metabolism in brain tissue might have been more strongly affected by hypertension in men, leading to a more substantial age-related memory decline in men compared with women.

The demonstrated association between serum creatinine and the initial stages of memory decline might be explained by the creatinine’s association with vagal activity in the elderly, which was described earlier [31]. Given that physiological vagal activity is a prerequisite for attention and working memory [32], the increased creatinine could be indirectly associated with attention and operative memory disturbance through mechanisms of autonomic nervous imbalance, especially in men. These mechanisms require further investigation as they could not be unambiguously concluded from the survey data.

We found that the 10th-90th percentile ranges of creatinine in all the subgroups of women were within the normal range. The 10th percentile for eGFRcr was slightly lower than the normal value (<60 mL/min/1.73 m^2^) in women who recalled two words, but the eGFRcr levels and the proportions of participants with kidney pathology showed no difference between women with varying word recall. In men, the 90th percentile for creatinine slightly exceeded the normal value in the group with 0–1 words recall, but not in the other subgroups, while the 10th percentile for eGFRcr was within the normal range in all the subgroups, and the proportions of participants with kidney pathology did not differ significantly. The de facto findings that the 90th percentile for the creatinine level exceeded the normal level in men with 0–1 words recall and that the 10th percentile for eGFRcr in women with 2 words recall was below the normal level are not evidence of the associations of these kidney function biomarkers with delayed memory function. However, they may indicate the initial signs of a reduced protein metabolic rate, which may have led to the decreased delayed memory function. A further increase in the creatinine level might have led to a renal failure, the accumulation of nitrogenous metabolism products, their toxic effects on the nervous tissue, and more substantial cognitive impairments [15,30].

According to our results, the 90th percentiles for uric acid levels in all men and women exceeded the upper physiological limit regardless of the number of words recalled. This indicates that there is no evidence for the association of uric acid with cognitive impairment. Complementary to that, a recent study demonstrated the neuroprotective effect of uric acid in the development of Alzheimer’s disease and dementia in Parkinson’s disease patients, while hypouricemia was found to be associated with the progression of these diseases [33]. Further investigation inti the role of uric acid in the development of memory disorders at the predementia stage requires a separate study.

Other authors have previously described a longitudinal association between reduced creatinine levels and cognitive impairment in the elderly at follow up [15]. In a prospective Finnish study of middle-aged residents without renal pathology, low creatinine levels were observed in men with low education, smoking, low physical activity, and lower indices of general cognitive function and working memory in particular. However, men with moderately elevated and normal creatinine levels had better associative and episodic memory than those with lower creatinine levels [14]. Similarly to our findings, the described association was shown in men only. The proximity of our results to the findings of prior research adds up to the evidence that a reduced serum creatinine and an increased GFR in older non-demented men may be indicative of the early stages of delayed memory impairments. An explanation for this finding could be the association of the relative decrease in creatinine with sarcopenia, which also has associations with mild cognitive impairment [34]. However, that is unlikely to be applicable in our study because the studied population sample included no sarcopenic patients.

The comparable creatinine and eGFRcr levels observed in men without delayed memory deficits (3 words recall) and with substantial impairment (0–1 words recall) are difficult to explain. It is possible that, older adults with substantial cognitive impairments receive more medical attention and are more likely to receive treatment for the somatic conditions that may cause the progression of cognitive decline, including kidney malfunctions. This hypothesis requires further investigation in longitudinal studies.

Defining the normal ranges of kidney function biomarkers for elderly people with sustained intrinsic capacity is methodologically challenging, especially in relation to the risk of cognitive impairments. In addition to creatinine levels, a solution must take into account the GFR levels, which are hyperbolically dependent on the creatinine level, as well as protein intake with food, and muscle mass volume [35]. The eGFRcr levels in the studied general population sample reflected sufficiently preserved filtration kidney function because the sample included only a small proportion of participants with chronic kidney disease and none at the dementia stage. This could have weakened the associations presented, but at the same time allows, considering the described ranges of kidney function biomarker levels as representative of a relatively healthy elderly population.

The described associations between renal function indices and delayed memory parameters may also be explained by the earlier described kidney–brain crosstalk mechanisms [36]. As the sample included no participants with medically ascertained renal insufficiency, the underlying mechanisms of the impaired homeostatic collaboration between brain and kidneys at the early stages of cognitive impairment could be the impaired sensitivity of baroreceptors and osmoreceptors and the associated impairments of blood vessel tonicity regulation. This may have conditioned the impaired microcirculation in the brain tissue and provoked disruption of molecular and cellular mechanisms of brain–kidney crosstalk, especially in men.

From a practical point of view, our findings reflect the importance of considering any significant changes in creatinine levels at a screening or medical observation of older people. Both decreases and increases, especially above 115 µmol/L, require an assessment of the risk of developing a cognitive impairment. As life expectancy has been growing in Russia’s north-western Federal District over the last two decades, and reached 73.1 years in 2022 [37], there is a growing burden of age-related cognitive disorders that must be addressed with early detection and treatment.

The climatic conditions of the European North of the Russian Federation entail region-specific risks for the development of cognitive disorders in elderly residents. Based on the Arkhangelsk region data, it was previously shown that almost half of the elderly people aged 60–74 years had age-associated disorders, and memory impairment was one of the leading geriatric syndromes [38]. Memory disorders have also been described in correlation with the lengthening of the time of cognitive visual evoked potentials P300 [39], and cognitive evoked auditory potentials P300 [40]. Findings from our and other studies outline the need for larger-scale research into the biochemical and electro-neurophysiological biomarkers of cognitive health in older adults.

A limitation of the study is the cross-sectional design, limiting the ability to draw conclusions about the nature of the described associations. The declines in kidney function might have caused memory declines, but a reverse causation cannot be ruled out. For example, participants with a reduced memory function could have had an impaired kidney function because of less healthcare contacts, having an underdiagnosed kidney disease, or forgetting to take proper medication. The relatively small sample size limited the statistical power to study and did not allow a deeper investigation into the discovered associations, for example, through stratification by age. The possibility of residual confounding cannot be excluded, although we attempted to take into account several factors potentially associated with both kidney function and cognitive impairment. Finally, the findings may not be generalizable beyond the geographic region of the study. Therefore, future studies with larger samples, enhanced consideration of potential confounders, and prospective designs are needed to further investigate the effects of kidney function on memory impairments and other cognitive disorders in older adults.

## 5. Conclusions

Serum creatinine level and glomerular filtration rate, respectively, had U-shaped and inverse U-shaped associations with delayed memory function in a general population sample of men 60–74 years with no dementia living in the European North of Russia. In particular, serum creatinine level was reduced in men with a minor delayed memory decline, but men with the lowest delayed memory performance had an elevated proportion of values exceeding the upper normal limit. Therefore, a reduced serum creatinine level in older men may indicate the initial stages of memory decline, while increased creatinine may reflect further stages of memory impairment. No associations between kidney function biomarkers and delayed memory were found in the analogue sample of women.

## Figures and Tables

**Table 1:** Socio-demographic, lifestyle, health, and cognitive characteristics of study participants according to sex.

Characteristics	Women *n* = 381	Men *n* = 262	*p* ^a^
Socio-demographic			
Age, M ± SD, years	66.9 ± 4.3	66.2 ± 4.3	0.032
Education, Abs (%)			0.053
- Higher (university)	114 (29.9)	90 (34.4)	
- Secondary professional (college)	229 (60.1)	134 (51.2)	
- Secondary (high school or lower)	38 (10.0)	38 (14.5)	
Lifestyle			
Smoking, Abs (%)			<0.001
- Never	326 (85.6)	69 (26.3)	
- Ex-smoker	36 (9.5)	136 (51.9)	
- Current	19 (5.0)	57 (21.8)	
Alcohol consumption, Abs (%)			<0.001
- Once a month or less	325 (85.3)	126 (48.1)	
- 2–4 times a month	50 (13.1)	81 (30.9)	
- 2–3 days a week and more often	6 (1.6)	55 (21.0)	
Health			
Body Mass Index, M ± SD, kg/m^2^	30.7 ± 5.8	28.1 ± 4.3	<0.001
Arterial hypertension ^b^, Abs (%)	325 (85.3)	204 (77.9)	0.015
Diabetes mellitus ^b^, Abs (%)	67 (17.6)	33 (12.6)	0.086
Liver diseases ^b^, Abs (%)	50 (13.2)	8 (3.1)	<0.001
Chronic kidney disease ^b^, Abs (%)	61 (16.0)	25 (9.5)	0.018
Cognitive function (MMSE)			
Predementia (scores 24–27), Abs (%)	124 (32.6)	105 (40.1)	0.050
Mistakes in the subtest assignments, Abs (%)			
- Time orientation	13 (3.4)	10 (3.8)	0.786
- Place orientation	50 (13.1)	43 (16.4)	0.244
- Immediate memory	4 (1.1)	2 (0.8)	0.710
- Attention and calculation	101 (26.5)	62 (23.7)	0.415
- Recall of three words (delayed memory)	207 (54.3)	176 (67.2)	0.001
- Speech	63 (16.5)	56 (21.4)	0.121
- Performing a three-stage command	35 (9.2)	24 (9.2)	0.991
- Reading and writing	48 (12.6)	50 (19.1)	0.025
- Copying	104 (27.3)	65 (24.8)	0.481

Abs—absolute number of participants; M—mean; SD—standard deviation; MMSE–Mini Mental State Examination. ^a^ *t*-Test for independent samples for continuous variables, Pearson’s χ^2^ test for categorical variables. ^b^ Doctor-diagnosed disease according to self-report.

**Table 2:** Socio-demographic, lifestyle, and health characteristics of women according to the number of words recalled.

Characteristics	Subgroup 1 3 Words Recall *n* = 174	Subgroup 2 2 Words Recall *n* = 158	Subgroup 3 0–1 Words Recall *n* = 49	*p* ^a^
***Socio-demographics***				
Age, M ± SD, yeas	66.5 ± 4.3	67.4 ± 4.2	67.2 ± 4.2	0.174
Education, Abs (%)				0.170
- Higher (university)	58 (33.3)	46 (29.1)	10 (20.4)	
- Secondary professional (college)	103 (59.2)	91 (57.6)	35 (71.4)	
- Secondary (high school or lower)	13 (7.5)	21 (13.3)	4 (8.2)	
***Lifestyle***				
Smoking, Abs (%)				0.276
- Never	146 (83.9)	135 (85.4)	45 (91.8)	
- Ex-smoker	21 (12.1)	12 (7.6)	3 (6.1)	
- Current	7 (4.0)	11 (6.9)	1 (2.0)	
Alcohol consumption, Abs (%)				0.422
- Once a month or less	151 (86.8)	130 (82.3)	44 (89.8)	
- 2–4 times a month	22 (12.6)	24 (15.2)	4 (8.2)	
- 2–3 days a week and more often	1 (0.57)	4 (2.5)	1 (2.0)	
***Health***				
Body Mass Index, M ± SD, kg/m^2^	30.5 ± 5.9	31.0 ± 5.9	30.2 ± 5.1	0.971
Arterial hypertension ^b^, Abs (%)	144 (82.8)	137 (86.7)	44 (89.8)	0.380
Diabetes mellitus ^b^, Abs (%)	32 (18.4)	25 (15.8)	10 (20.4)	0.710
Liver diseases ^b^, Abs (%)	25 (14.4)	21 (13.3)	4 (8.2)	0.523
Chronic kidney disease ^b^, Abs(%)	27 (15.5)	28 (17.7)	6 (12.2)	0.640

Abs—absolute number of participants; M—mean; SD—standard deviation. ^a^ ANOVA for continuous variables, Pearson’s χ^2^ test for categorical variables. ^b^ Doctor-diagnosed disease according to self-report.

**Table 3:** Socio-demographic, lifestyle, and health characteristics of men according to the number of words recalled.

Characteristics	Subgroup 1 3 Words Recall *n* = 86	Subgroup 2 2 Words Recall *n* = 114	Subgroup 3 0–1 Words Recall *n* = 62	*p* ^a^
***Socio-demographics***				
Age, M ± SD, yeas	65.7 ± 4.4	66.4 ± 4.3	66.4 ± 3.9	0.517
Education, Abs (%)				0.942
- Higher (university)	28 (32.6)	42 (36.8)	20 (32.3)	
- Secondary professional (college)	44 (51.1)	57 (50.0)	33 (53.2)	
- Secondary (high school or lower)	14 (16.3)	15 (13.2)	9 (14.5)	
***Lifestyle***				
Smoking, Abs (%)				0.868
- Never	22 (25.6)	30 (26.3)	17 (27.4)	
- Ex-smoker	46 (53.5)	56 (49.1)	34 (54.8)	
- Current	18 (20.9)	28 (24.6)	11 (17.7)	
Alcohol consumption, Abs (%)				0.746
- Once a month or less	40 (46.5)	56 (49.1)	30 (48.4)	
- 2–4 times a month	31 (36.1)	32 (28.1)	18 (29.0)	
- 2–3 days a week and more often	15 (17.4)	26 (22.8)	14 (22.5)	
***Health***				
Body Mass Index, M ± SD, kg/m^2^	27.8 ± 4.2	27.8 ± 4.4	29.1 ± 4.1	0.971
Arterial hypertension ^b^, Abs (%)	66 (76.7)	82 (71.9)	56 (90.3)	0.019
Diabetes mellitus ^b^, Abs (%)	7 (8.1)	16 (14.0)	10 (16.1)	0.291
Liver diseases ^b^, Abs (%)	4 (4.8)	1 (0.89)	3 (4.8)	0.199
Chronic kidney disease ^b^, Abs(%)	11 (12.8)	7 (6.1)	7 (11.3)	0.247

Abs—absolute number of participants; M—mean; SD—standard deviation. ^a^ ANOVA for continuous variables, Pearson’s χ^2^ test for categorical variables. ^b^ Doctor-diagnosed disease according to self-report.

**Table 4:** MMSE scores and kidney function biomarkers in women according to the number of words recalled.

Parameter	Subgroup 1 3 Words Recall *n* = 174	Subgroup 2 2 Words Recall *n* = 158	Subgroup 3 0–1 Words Recall *n* = 49	*p* ^a^	*p* _linear_ _trend_ ^b^	*p* _quadratic_ _trend_ ^c^	Normal Range
	Median [10p–90p]						
MMSE, total score	29.0 [26.0–30.0]	28.0 [25.0–29.0]	27.0 [25.0–28.0]	<0.001	<0.001	0.214	25.0–30.0
Creatinine, μmol/L ^d^	69.5 [61.0–85.0]	71.0 [60.0–91.0]	70.0 [62.0–80.0]	0.721	0.965	0.346	50.4–110.5
Uric acid, mg/dL	5.0 [3.7–7.4]	5.2 [3.6–7.3]	5.0 [3.6–7.4]	0.831	0.649	0.596	2.6–6.0
eGFRcr, ml/min/1.73 m^2 d,e^	82.1 [64.7–95.1]	78.4 [58.1–93.9]	81.8 [67.5–93.7]	0.649	0.981	0.278	≥60.0

10p—10th percentile; 90p—90th percentile; MMSE—Mini Mental State Examination; eGFRcr—estimated glomerular filtration rate based on the serum creatinine concentration. ^a^ ANCOVA with adjustment for age. ^b^ Multiple linear regression with subgroup number entered as a continuous independent variable and with adjustment for age. ^c^ Similarly specified multiple linear regression with quadratic term additionally entered. ^d^ Comparisons were made and trends estimated using *ln*-transformed dependent variable values. ^e^ The values were calculated using the CKD-EPI Creatinine Equation (2021).

**Table 5:** MMSE scores and kidney function biomarkers in men according to the number of words recalled.

Parameter	Subgroup 1 3 Words Recall *n* = 86	Subgroup 2 2 Words Recall *n* = 114	Subgroup 3 0–1 Words Recall *n* = 62	*p* ^a^	*p* _linear_ _trend_ ^b^	*p* _quadratic_ _trend_ ^c^	Normal Range
	Median [10p–90p]						
MMSE, total score	29.0 [26.0–30.0]	28.0 [26.0–29.0]	27.0 [25.0–28.0]	<0.001	<0.001	0.927	25.0–30.0
Creatinine, μmol/L ^d^	87.0 [73.0–109.0]	82.0 [69.0–101.0]	87.0 [75.0–115.0]	0.021	0.828	0.002	50.4–110.5
Uric acid, mg/dL	6.0 [4.4–8.0]	6.1 [4.5–8.1]	6.0 [4.7–8.0]	0.695	0.728	0.721	3.5–7.2
eGFRcr, ml/min/1.73 m^2 d,e^	84.4 [63.4–98.1]	92.0 [68.5–99.1]	84.0 [60.0–97.1]	0.045	0.796	0.005	≥60.0

10p—10th percentile; 90p—90th percentile; MMSE—Mini Mental State Examination; eGFRcr—estimated glomerular filtration rate based on the serum creatinine concentration. ^a^ ANCOVA with adjustment for age and hypertension. ^b^ Multiple linear regression with subgroup number entered as a continuous independent variable and with adjustment for age and hypertension. ^c^ Similarly specified multiple linear regression with quadratic term additionally entered. ^d^ Comparisons were made and trends estimated using *ln*-transformed dependent variable values. ^e^ The values were calculated using the CKD-EPI Creatinine Equation (2021).

## Data Availability

The ESSE-RF3 data are accessible for research purposes. The inquiries are to be sent to esserf2020@gmail.com. All data requests are considered by the study team, guided by national regulations for protecting of personal information, confidentiality agreement with participants, and their informed consents.

## References

[1] Zakharov V.V. (2005). Age-related disorders of memory and attention. Cons. Medicum.

[2] Levin O.S. (2019). Predementia neurocognitive impairment in the elderly. Zh Nevrol. Psikhiatr Im. S. S. Korsakova.

[3] Nasreddine Z.S., Phillips N.A., Bédirian V., Charbonneau S., Whitehead V., Collin I., Cummings J.L., Chertkow H. (2005). The Montreal Cognitive Assessment, MoCA: A brief screening tool for mild cognitive impairment. J. Am. Geriatr. Soc..

[4] Ritter A., Hawley N., Banks S.J., Miller J.B. (2017). The Association between Montreal Cognitive Assessment Memory Scores and Hippocampal Volume in a Neurodegenerative Disease Sample. J. Alzheimers Dis..

[5] Tsoi K.K., Chan J.Y., Hirai H.W., Wong S.Y., Kwok T.C. (2015). Cognitive Tests to Detect Dementia: A Systematic Review and Meta-analysis. JAMA Intern. Med..

[6] Pangman V.C., Sloan J., Guse L. (2000). An Examination of Psychometric Properties ofthe Mini-Mental Status Examination and the Standardized Mini-Mental Status Examination: Implications for Clinical Practice. Appl. Nurs. Res..

[7] Arevalo-Rodriguez I., Smailagic N., Roqué-Figuls M., Ciapponi A., Sanchez-Perez E., Giannakou A., Pedraza O.L., Cosp X.B., Cullum S. (2021). Mini-Mental State Examination (MMSE) for the early detection of dementia in people with mild cognitive impairment (MCI). Cochrane Database Syst. Rev..

[8] Price C.C., Garvan C., Hizel L.P., Lopez M.G., Billings F.T. (2017). Delayed Recall and Working Memory MMSE Domains Predict Delirium following Cardiac Surgery. J. Alzheimers Dis..

[9] Orock A., Logan S., Deak F. (2020). Age-Related Cognitive Impairment: Role of Reduced Synaptobrevin-2 Levels in Deficits of Memory and Synaptic Plasticity. J. Gerontol. A Biol. Sci. Med. Sci..

[10] Cho Y.H., Kim G.H., Park J.J. (2021). Mitochondrial aconitase 1 regulates age-related memory impairment via autophagy/mitophagy-mediated neural plasticity in middle-aged flies. Aging Cell.

[11] Nagibovich O.A., Shipilova D.A., Shchukina N.A., Trandina A.E. (2020). Problems of quantitative estimation of excretory kidney function based on creatinine. Nephrology.

[12] Diago C.A.A., Señaris J.A.A. (2020). Should we pay more attention to low creatinine levels? ¿Debemos prestar más atención a la creatinina baja?. Endocrinol. Diabetes Nutr..

[13] Papadopoulou S.K., Voulgaridou G., Kondyli F.S., Drakaki M., Sianidou K., Andrianopoulou R., Rodopaios N., Pritsa A. (2022). Nutritional and Nutrition-Related Biomarkers as Prognostic Factors of Sarcopenia, and Their Role in Disease Progression. Diseases.

[14] Hakala J.O., Pahkala K., Juonala M., Salo P., Kähönen M., Hutri-Kähönen N., Lehtimäki T., Laitinen T.P., Jokinen E., Taittonen L. (2022). Repeatedly Measured Serum Creatinine and Cognitive Performance in Midlife: The Cardiovascular Risk in Young Finns Study. Neurology.

[15] Etgen T., Sander D., Chonchol M., Briesenick C., Poppert H., Förstl H., Bickel H. (2009). Chronic kidney disease is associated with incident cognitive impairment in the elderly: The INVADE study. Nephrol. Dial. Transplant..

[16] Istomin A.V., Fedina I.N., Shkurikhina S.V., Kutakova N.S. (2018). Food and the North: Hygienic problems of the Arctic zone of Russia. Gig. I Sanit..

[17] Bichkaeva F.A., Bichkaev A.A., Volkova N.I., Vlasova O.S., Tretyakova T.V., Shengoff B.A. (2015). Modulating effect of biogenic amines, insulin and cortisol on protein metabolism in residents of various climatic and geographical territories. Vestn. North. (Arct.) Fed. Univ. Ser. Med. Biol. Sci..

[18] World Health Organization (2015). WHO World Report on Ageing and Health.

[19] Zhou Y., Ma L. (2022). Intrinsic Capacity in Older Adults: Recent Advances. Aging Dis..

[20] George P.P., Lun P., Ong S.P., Lim W.S. (2021). A Rapid Review of the Measurement of Intrinsic Capacity in Older Adults. J. Nutr. Health Aging.

[21] Drachev S.N., Popov V.A., Simakova A.A., Gorbatova M.A., Kudryavtsev A.V., Shagrov L.L., Popova D.A., Grjibovski A.M., Kontsevaya A.V., Yushmanova T.N. (2022). Study profile: Oral health assessment among participants of “Epidemiology of cardiovascular diseases in Russian regions. Third study” in the Arkhangelsk region. Ekol. Cheloveka Hum. Ecol..

[22] Krieger E., Sharashova E., Kudryavtsev A.V., Samodova O., Kontsevaya A., Brenn T., Postoev V. (2023). COVID-19: Seroprevalence and adherence to preventive measures in Arkhangelsk, Northwest Russia. Infect. Dis..

[23] Creavin S.T., Wisniewski S., Noel-Storr A.H., Trevelyan C.M., Hampton T., Rayment D., Thom V.M., Nash K.J., Elhamoui H., Milligan R. (2016). Mini-Mental State Examination (MMSE) for the detection of dementia in clinically unevaluated people aged 65 and over in community and primary care populations. Cochrane Database Syst. Rev..

[24] National Kidney Foundation (2021). CKD-EPI Creatinine Equation. https://www.kidney.org/content/ckd-epi-creatinine-equation-2021.

[25] National Kidney Foundation What Is the Criteria for CKD. https://www.kidney.org/professionals/explore-your-knowledge/what-is-the-criteria-for-ckd.

[26] Petersen R.C. (2016). Mild cognitive impairment. Contin. Lifelong Learn. Neurol..

[27] Wolfova K., Creese B., Aarsland D., Ismail Z., Corbett A., Ballard C., Hampshire A., Cermakova P. (2022). Gender/Sex Differences in the Association of Mild Behavioral Impairment with Cognitive Aging. J. Alzheimers Dis..

[28] Overton M., Pihlsgård M., Elmståhl S. (2019). Prevalence and Incidence of Mild Cognitive Impairment across Subtypes, Age, and Sex. Dement. Geriatr. Cogn. Disord..

[29] Miyawaki C.E., Liu M. (2019). Gender differences in cognitive impairment among the old and the oldest-old in China. Geriatr. Gerontol. Int..

[30] Fomina N.V., Egorova M.V. (2016). Features of cognitive impairment in patients with chronic kidney disease depending on the presence of arterial hypertension. Nephrology.

[31] Protasov K.V., Dzizinsky A.A. (2010). Heart rate variability and renal function in elderly and senile patients with arterial hypertension. Sib. Med. Zh..

[32] Sun L., Peräkylä J., Holm K., Haapasalo J., Lehtimäki K., Ogawa K.H., Peltola J., Hartikainen K.M. (2017). Vagus nerve stimulation improves working memory performance. J. Clin. Exp. Neuropsychol..

[33] Kunitskaya N.A., Ariev A.L. (2022). The role of hyperuricemia in the development of cognitive changes in the elderly. Adv. Gerontol..

[34] Cabett Cipolli G., Sanches Yassuda M., Aprahamian I. (2019). Sarcopenia Is Associated with Cognitive Impairment in Older Adults: A Systematic Review and Meta-Analysis. J Nutr Health Aging..

[35] Delanaye P., Cavalier E., Pottel H. (2017). Serum Creatinine: Not So Simple!. Nephron.

[36] Afsar B., Sag A.A., Yalcin C.E., Kaya E., Siriopol D., Goldsmith D., Covic A., Kanbay M. (2016). Brain-kidney cross-talk: Definition and emerging evidence. Eur. J. Intern. Med..

[37] Federal State Statistic Service Life Expectancy at Birth by Regions of the Russian Federation for 2022. https://eng.rosstat.gov.ru.

[38] Popov V.V., Novikova I.A., Trokhova M.V., Litvyakova M.L., Kudinova A.V. (2019). Early diagnosis and prevention of age-related disorders in elderly and senile people living in the European North of Russia. Russ. J. Prev. Med..

[39] Deryabina I.N., Dzhos Y.S. (2017). Characteristics of the cognitive evoked potentials in elderly people with cognitive decline. Adv. Gerontol..

[40] Poskotinova L., Khasanova N., Kharak A., Krivonogova O., Krivonogova E. (2023). Parameters of Auditory Evoked Related Potentials P300 in Disorders of Different Cognitive Function Domains (Visuospatial/Executive and Memory) in Elderly Hypertensive Persons. Diagnostics.

